# Primary Health Care: Comparing Public Health Nursing Models in Ireland and Norway

**DOI:** 10.1155/2013/426107

**Published:** 2013-03-31

**Authors:** Anne Clancy, Patricia Leahy-Warren, Mary Rose Day, Helen Mulcahy

**Affiliations:** ^1^Department of Health and Social Work, School of Nursing, Harstad University College, 9480 Harstad, Norway; ^2^University of Tromsø, 9019 Tromso, Norway; ^3^School of Nursing & Midwifery, Brookfield Health Sciences Complex University College Cork, Cork, Ireland

## Abstract

Health of populations is determined by a multitude of contextual factors. Primary Health Care Reform endeavors to meet the broad health needs of populations and remains on international health agendas. Public health nurses are key professionals in the delivery of primary health care, and it is important for them to learn from global experiences. International collaboration is often facilitated by academic exchanges. As a result of one such exchange, an international PHN collaboration took place. The aim of this paper is to analyse the similarities and differences in public health nursing in Ireland and Norway within the context of primary care.

## 1. Introduction

The movement toward primary care as a model of health care service delivery was introduced 30 years ago and has been moving in that direction since then. It has been reiterated across international policy documents during this period [1]. The time for prevarication has passed and action is warranted. Public health nurses (PHNs) due to their public health orientation and guiding philosophy are acutely sensitive to any proposed changes in health policy underpinned by primary health care [2]. This is due to the fact that they work in the community and provide universal low threshold services guided by health promotion and disease prevention and their health outcomes are difficult to measure. Evidence indicates that a preventative approach to community-based health interventions reduces the use of acute hospital services, improves the management of chronic illnesses, and empowers clients to self-care [3]. The remit of PHNs encompasses nursing and public health; therefore, the focus is on primary, secondary, and tertiary prevention [4]. The aim of this paper is to discuss primary health and primary health care and analyse similarities and differences between Ireland and Norway in relation to geography, demography, and health status. The origins of public health nursing are presented. This is followed by an exploration of the different models and the merits and demerits of specialists and generalists' roles and functions in both countries. The paper concludes by pulling together the salient points contributing to a greater insight to PHN practice in Ireland and Norway. The impetus for this paper came from an academic collaboration between the authors as a result of one authors' (AC) Erasmus visit to University College Cork in 2012. The European Erasmus programme promotes educational exchanges between university students and staff. Ireland and Norway are participants in this programme. This visit presented an ideal opportunity to examine the similarities and differences of public health nursing and primary care in two jurisdictions. This paper will contribute to the discourse on public health nursing in the context of primary health care internationally.

## 2. Primary Health Care and Primary Care

Primary health care (PHC) as defined by the WHO in 1978 [5] is essential health care based on practical, scientifically sound and socially acceptable methods. Primary health care is considered to be both a philosophy and an approach to providing health resources. The approach is usually termed primary care (PC) and in Ireland is often used synonymously with “general practice” (GP). However, whilst PC incorporates GP care, it encompasses a wide range of health and personal social services delivered by a variety of professionals and is seen as a first point of contact service [6]. Countries with more highly developed systems of primary health care tend to have lower health care costs. Norway was one of the first countries to adopt this model of health care. The organisation of primary care in Norway is decentralised to municipalities. In 1984, 430 local authorities were made responsible for financing and providing primary care services founded on social democratic values and funded by taxes and block grants [7]. It is much easier to support PHC reforms when growth in health expenditure is through prepaid systems than out of pocket expenditures [2].

In contrast, in Ireland Primary care was first proposed as a model of health care to be considered in the mid-1980s [8] but due to the poor fiscal economy, in essence, the first primary care strategy was not published until 2001. This strategy established a community-driven model designed to strengthen the capacity of services at primary care level. As a consequence, dependency on secondary can be minimized achieving increased accessibility to local primary care teams (PCTs). 

Health care in Ireland is a two-tier system where public and private sectors exist. The public health care system is governed by the Health Act of 2004 [9], which established the Health Service Executive to be responsible for providing health and personal social services to everyone living in Ireland. The public health system, despite massive expenditure in recent years, has a number of on-going issues which could have an impact on primary care services. These include long waiting lists; over capacity on hospital beds; patients awaiting admission on trolleys in the A&E departments; moratorium on staff recruitment and staff shortages. Ireland's two-tier health care system has failed in many respects to delivery adequate, fair, and equitable services to meet people's needs [10]. Not all citizens in Ireland have free health care at the point of delivery as it is based on income. Many health care payment schemes operate such as the General Medical Services (GMS) card, Pay Related Social Insurance (PRSI), and drug payment scheme. About 39% of the population are covered by a medical card or a GP visit card [11]. In general, PHNs in Ireland deal with all children and adults with GMS. Eligibility for non-GMS adults is contentious but PHNs deal with these referrals on a case by case basis [12]. 

The reality of the number and composition of primary care teams in Ireland has yet to be realised. Approximately 600–1,000 primary care teams (PCTs) were envisaged by the primary care strategy to meet population needs [13]. Data from the Comptroller & Auditor General [14] suggest that there were 319 PCTs and 24 new primary care centres; however, Gartland [15] reported a figure of 411 teams, and a survey of general practitioners (GPs) reported that only 36% were part of a functioning PCT [16]. In Norway, the compositions of the PCTs are similar to Ireland with regard to the health care professionals involved. The large number, small size, and organisational models of Norwegian municipalities necessitate flexibility and intermunicipal collaboration in the smallest communities in order to provide functional interdisciplinary PCTs. As in Ireland, GPs work in private practices but are contracted by the municipalities to perform public health services. PHNs provide domiciliary home visiting services to all newborns and work mainly at health clinics and school health services, whereas in Ireland, PHNs are generalists with some specialism within child health. In Ireland, 22% of areas have a dedicated school PHN [12]. Norway is currently in the throes of a new major public health reform (Act of 2011) with the primary focus on prevention and early intervention [17, 18] and governed from local municipalities. A consequence of the new reform [18] with shorter hospital stays for mothers and babies after childbirth could influence their workload. The health system in Ireland, which is governed centrally by the Health Service Executive, is also under reform. This reform also includes a move towards universal health insurance which envisages equitable access to health services. Early hospital discharge in both countries has the potential to increase the need for PHNs services.

Norway is more advanced in their health reforms, and devolution of care in the community locally within a team is a key component of PC. However, there are challenges in maintaining professional individuality so that the clinical accountability of professions is not lost. The individual contribution of distinct professions to the team decision needs to be transparent. Reports to the government on public health work focus on coordination of services and not on professional groups [18, 19], so that now the focus is even more so on professional neutrality. Due to the nature of preventive and promotive work, it is difficult to measure the effects of PHNs' work. Aging populations and lack of visibility of public health nursing in official documents provide challenges. In report no. 16, (Stortingsmelding, 2002-2003) the role of professions is toned down. This is illustrated by the following quote: “it's important to focus on what has to be done in the municipality—not on who does it” [19]. Stenvoll et al. [20] compare this report with a similar public health document from 1993 and conclude that the focus on preventive institutions, such as child health clinics and school health services as well as professions working there, has been weakened. The trend towards professional neutrality is reiterated across current government reports. Reducing the visibility of public health nursing is not conducive to professional development and can mitigate against effective primary care. Effective primary care is more dependent on the context of care than the composition of teams. It ultimately reflects on all the determinants of health. Therefore, it is necessary to examine the health contexts of both Ireland and Norway.

## 3. Ireland and Norway: Geography, Demography, and Health 

Ireland and Norway differ in geography and economic situation but have some similarities in relation to population statistics and public health challenges. Demography and vital statistics for Ireland and Norway are presented in Table 1.

 Ireland is often called the “Emerald Isle.” The country is characterised by vibrant green fields, hedgerows, low plains, rugged coastlands, lakes, rivers, and islands. Climate in Ireland is mainly mild and humid, winter days are drizzly, cold, and short because of the Gulf Stream, and there is rarely snow. However artic conditions and snow in 2010 presented major challenges in Ireland for the delivery of health services. On the contrary, Norway is dominated by mountainous or high terrain, and the country is renowned for its Viking heritage, natural resources, and its long indented coastline and fjords. Climate and geography create challenges for an egalitarian provision of municipal health services and being a welfare state means, inequalities are less acceptable in Norway. The climate in the country differs from North to south, but winters are cold throughout. 

Ireland is a member of the European Union (EU), is the third largest island in Europe, and is situated to the North West of Continental Europe. Politically Ireland is divided between the Republic of Ireland (ROI) (26 counties) and Northern Ireland (6 counties) which is part of the United Kingdom. Ireland has a population of 4,588,252 people; 1.2 million people live in Dublin city and county and the density of population is approximately 64.95 people per square km [21]. In Ireland, 535,393 people are aged over 65 years, but life expectancy is slightly lower for females 81.6 years and for men 76.8 years [22]. Overall the population of Ireland is relatively young, and birth rates are 15.81 births/1,000 population. Conversely Norway is not a member of the EU and borders on Russia, Sweden, and Finland. It has a similar population size of approx. 4.8 million but in contrast to Ireland, it is one of the most sparsely populated countries in Europe with only 15 inhabitants per square km. Most of the municipalities are small, and a quarter of the population lives in rural areas. Twenty-six percent of Norway's population is under 20 years of age, and birth rates are 12.1 births/1,000 population. There are 742,000 aged over 65 years in Norway, and life expectancy for females in Norway is 83.4 years and 79 years for men and these rank among the highest in the world [23]. In terms of population structure, Ireland's population is younger and still growing, whereas Norway's has stabilised and their life expectancy is much greater. 

The social, economic, and environmental conditions in which people live strongly influence health. There is a strong association between environment, ill health, chronic illness, and morbidity [24]; and Ireland has many health inequalities. For example, the life expectancy for the travellers (Ireland's main minority ethnic group) is currently only 61.7 years consistent with life expectancy of general population in the 1940s in Ireland [25]. Not surprisingly, they were also less likely to report good health. Poverty levels are increasing and in 2010, 15.8% of the population (706,500) had incomes below €10,831 [26]. Ireland has the highest proportion of children in the EU (24.5%) [24], nearly 9% of these children live in families in consistent poverty and over 18% are at risk of poverty [27]. Mental health, suicide, and poor physical health and well-being are significantly higher in lower social classes and socially deprived areas [28]. The first longitudinal study on people aged over 50 years found that older adults have excellent health. However, it also found that those unemployed had poorer health [29]. There is a growing epidemic of obesity levels in both younger and older people [22, 30, 31]. Ireland has low breast feeding rates, and the rates are more pronounced in lower socioeconomic groups. This is also the case in relation to low birth weight [32]. Current health services in Ireland favour the more well off, yet people who are less well off and socially excluded have poorer health and thus may be more in need of services [33].

Conversely Norway is one of the richest countries in the world, and there has been no increase in poverty in recent years [34]. There are fewer poor people in Norway compared with other countries, and poverty seems to be a temporary condition for most people. Immigrants from nonwestern countries are those most affected. The extensive nature of public welfare services in Norway ensures that poor people are seldom deprived of necessary living conditions [34]. Money can ensure the provision of services but cannot buy health, and Norway has a widening gap in issues of inequalities in health [35]. Norway has topped the UN's annual ranking for national achievement in health, education, and income [36]. The general health of population in Norway is good but there is still a sizeable gradient in morbidity and mortality [37]. It is well recognized that early childhood years can directly and indirectly affect health in later life, and children's living conditions are closely linked to their family's socioeconomic status [38]. 

Norwegian health services experience many challenges in relation to aging populations, shortened hospital stay, heightened expectations, and an increasing dependency on expertise to solve problems [39]. Psychological problems are a major challenge for public health in Norway [19], and every third adolescent that is in touch with a Norwegian PHN has psychological problems [40]. Gambling addiction often coincides with other health and social disorders, and excessive on-line gaming amongst young people provides new challenges for their health and well-being. One in four pupils in Norway who start secondary education drops out and this can impact on health and life expectancy [38]. Conversely more recent data reported that more children in Ireland leave school early than children in Norway [41], and there is greater socio-economic gradient. There is an increase in reports of teenage suicides and some of these have been linked to cyber bullying, which makes the health needs of adolescents more of a public health priority [42]. 

Certain population groups in Norway have special health challenges such as those with long-term social problems, people living alone, immigrants, people with mental health issues, and children and young people at risk. Research has shown that ethnic groups in Norway have suffered ethnic discrimination [43]. Similarly Ireland faces many public health challenges in relation to health inequalities, health, aging populations, chronic illness, medical advances, shorter hospital stay, social factors (living alone, isolation, and poor social networks), and economic decline [22, 25]. Both Ireland and Norway are experiencing significant public health challenges in relation to growing levels of chronic illness-related to lifestyle factors [30, 38]. The health challenges of susceptible groups are relevant to PHNs' work in the context of primary health care in both Ireland and Norway. 

## 4. Public Health Nursing in Norway and Ireland

Community nurses in Norway were traditionally concerned with caring for the sick. School health services were introduced with the implementation of the School Act in 1860 [44], and the first mother and child health clinic was opened in 1911 [45]. The early development of Norwegian public health nursing services was influenced by the American model. In many communities, the public health nurse and doctor were the only public health professionals until the late seventies. 

Norwegian PHNs are nurses with a specialist qualification in public health nursing. Their current tasks do not involve nursing care of the sick, that is, curative nursing. This care is provided by district nursing services and nurses in local institutions. PHNs in Norway are usually assigned a geographical area and provide universal services at child health clinics and school health services. They perform home visits and carry out immunisations and developmental screening; they also counsel and give advice to individuals and groups. Almost 100% of families avail of the services at child health clinics. There are 2069 PHNs employed in municipal family health clinics and school health services [46], and in Ireland there were 1702 PHNs employed in the Irish Health Service Executive [47].  Table 2 illustrates key similarities and differences related to education, organisational structure, remit, focus of care, and current challenges in public health nursing in Ireland and Norway.

PHNs in Ireland operate at the level of generalist nurses with a specialist qualification in public health nursing. Public Health Nursing similarly originated in the 1800s and because of historical links with Britain mirrored developments there. The origins of the service were more specialist in orientation, that is, district nursing and community midwifery. The model was specifically generalist since the 1960s, and this was recognised as a strength by the Commission on Nursing [48] who recommended a continuation of generalist geographic focus. However, there has always been an acknowledgement of the specialist versus generalist debate in community nursing. A number of reviews have taken place in Ireland, the most recent [12] of which reiterates the need to reexamine the organisational model for reform. In Norway, the tendency has been to move towards a specialised role in providing services for families and the young population, which will be discussed further in the next section.

Smith describes public health nursing as a nursing speciality that combines nursing and public health principles [49]. The individual-/family-based approach is the Norwegian PHNs' strength, and PHNs have been criticised for not becoming more engaged in public health work at a community level [50]. Helseth [51] explicates, however, the continued importance of the PHN's direct contact with individuals and groups. Primary preventative child health work is carried out mainly by physicians in the USA and mainly nurses in Western Europe, including Ireland and Norway. Eliciting and attending to parental concerns is a key element of effective developmental surveillance and is in line with international best practice [52]. It is acknowledged that there are significant gains from home visiting [53] and sound reasons for the service to remain universal. Universal and targeted child health intervention programs have been shown to improve maternal and child health and reduce inequalities in health [38]. There is a continued need for a universal service that identifies and facilitates the health needs of ordinary people [54, 55]. Specific measures of service effectiveness may be lacking but in terms of efficiency in the delivery of core health checks Irish PHNs have achieved an adherence rate of between 81 and 97% with the scheduled developmental checks [56]. The primary immunisation programme which is actively promoted by PHNs achieves uptake rates over 90% in all of the reporting districts and rates of 95% in 75% of reporting districts [57]. Similarly, immunisation coverage for children in Norway is between 92 and 95% (regardless of socio-economic groupings) with an increase from 2010 [58]. Information technology has provided Norway with an efficient national immunisation registry (SYSVAK) [58]. In one small area, good public health outcomes are being achieved in both countries. However, population outcome data is limited and is not conducive to promoting the reform of the PHN role. In contrast, public health measures have been far more successful in Norway, which has high breastfeeding initiation rates of 99% and duration 80% at 6 months [59]. Ireland has been less successful with an initiation of 46% and duration of 13% at 6 months [60]. Quality and accountability in primary care have been compromised by relatively poor investment in health care informatics and technology in Ireland [61]. 

 Reform is contingent on the implementation of the recommendations of the National Health Information Strategy [62] to implement electronic health records and unique health identifier numbers. This is a particular requirement where child health is concerned.

Bellman and Vijeratnam [63] caution that the benefits of developmental surveillance should not only be viewed in terms of the abnormalities detected, but also in terms of the support and reassurance to parents. Public health nursing services focus on health promotion and the provision of supportive counselling services. Supportive counselling provided by PHNs has been shown to be effective [64–66]. The focus in Ireland and Norway is also on disease prevention through immunisation programs, developmental screening, and subsequent referrals to other services. Use of specialized health services by children and young people increases with the length of parents education, whereas use of PHNs primary care health services at clinics and schools is more determined by need than social status [38]. Reasons for social inequalities in health can start in childhood; each individual factor may not be important but when these social factors are added up their negative effects can be significant [67].

 The school health service has insufficient capacity in many of Norway's municipalities [38]. Not all children and adolescents receive adequate psychological care. It has been put forward that PHNs lack competencies in providing mental health services for this group [40]. Norway is currently concerned with providing specialised mental health services for children, young people, and families in their own communities [18], an area that is very underresourced in Ireland [68]. Many children with mental health problems need assistance from several services, and collaboration is vital. Psychosocial problems are an important focus for PHNs and there is a need for improved collaboration with PHNs on psychosocial, medical, and child protection issues [50, 51, 69, 70]. A recent Norwegian national survey has shown that mental health services are those missed most by communal primary care professionals [71]. Emotional and mental health care in schools is not a feature of the work of PHNs in Ireland where the focus is on immunisations and screening for vision, hearing, and growth [12]. Although emotional health is acknowledged as being vitally important by the HSE [72], it was found by the ONMSD [12] that the school immunisation programme takes precedence over this and indeed there are also unmet targets in relation to screening.

**Figure 1:**
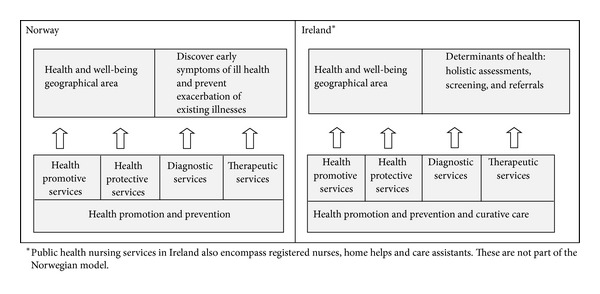
Adapted health promotive and preventive action model illustrating current range of services provided by PHNs in Ireland and Norway.

Elo and Calltorp [73] developed a *health promotive and preventive action model* (HPA model) for illustrating the wide range of public health services provided by PHNs. The model was constructed in order to illustrate wherein the process of health-ill health and at what developmental stages PHNs provide health care services. An adapted HPA model (Figure 1) is used to illustrate current Norwegian and Irish public health nursing practice related to the health-ill health continuum. Norwegian PHNs' services for children and young people can be described as being health promotive, supportive, health protective, diagnostic, and therapeutic. The Irish PHNs generalist services include curative care and encompass other services not included in the Norwegian PHNs remit. All models have limitations. The HPA model provides a framework and cannot capture all factors that influence public health nursing service provision.

## 5. Advancing Public Health Nursing

The previous three sections explored how public health nursing in Ireland and Norway has evolved. However, its not as if future PHN service delivery models were not previously considered. For example, there has always been an acknowledgement of the specialist versus generalist debate that exercises those involved in community health nursing. The journal Public Health Nursing republished an article [74] that first appeared in 1916. This article has as a core message that the debate should not be specialist *or* generalist; rather the model should be specialist *and* generalist. Brainard [74] recognised the fact that communities have different complexities and requirements which she believed should ultimately determine the best model and that there is a place and need for both models. She used the example of general practitioner and medical specialist to argue that they supplement each other's work rather than duplicating it.

McKenna et al. [75] were the only authors to study professional and lay views of generic and specialist roles in the island of Ireland. Each jurisdiction in Ireland has very different models of nursing in the community, that is, 11 different specialist community nurses in Northern Ireland (NI) and one generalist PHN in the Republic of Ireland (ROI). Although there are not as many specialist nurses in the community, public health nursing in Norway has a long tradition of specialist practice and thus is like NI in that respect. It would appear that Norway has achieved a good specialist/generalist balance in terms of community nursing. McKenna et al.'s [75] study concluded with the view that there were too many specialists in NI and too few in the ROI, and both were “heading for an imbalance” (page  544). 

In the ROI it would appear that the day of imbalance has arrived as there have been recent moves to seriously consider moving public health nursing in a specialist direction [12, 47]. In response to the problem of “duplication of effort” identified by the Office of the Nursing & Midwifery Services Director [12] and the Institute of Community Health Nursing [76]; it was recommended that “consideration must be given to matching skills with the health needs of the population in a more integrated manner” (page  19). A more pressing imperative comes in the wake of a number of child protection reports which highlighted that child welfare and family needs were not prioritised. The current generalist role of PHNs is seen as serious disadvantage from a child and family perspective as the curative role constantly takes precedence [47]. This National report [47] indicated that illness-related nursing care was prioritised over child health and welfare. 

The Minister for Children and Youth Affairs [47], in the task force report document, recommends that the PHNs who provide the child and family part of the service should be directly employed by the Child and Family Support Agency (CFSA). Efforts to avoid fragmenting the service could be achieved by colocating PHNs with the local health service. The precise detail of how this change in governance would be configured has yet to be explored. However, the ICHN has canvassed the views of their members in relation to four potential options and found broad support for the need to change the current method of service delivery [76]. 

McKenna et al. [75] found that while more specialist nurses are required in the community in the future, this has the potential to increase role conflict between nurses and other community professionals. This issue was raised previously for Norway which faces similar challenges regarding coordination and collaboration. Nevertheless, it is suggested by McKenna et al. [75] that colocation of professionals from different organisations can create an arena for staff to work across professional boundaries, to recognise their joint role as supportive professionals, and thus to enable families to find their way through the challenges they face [55, 69]. 

A further area of concern in Ireland is the schools service provided by PHNs. According to the ONMSD [12], there is a general lack of direction and focus in the school health programme. Local health office areas vary in relation to whether or not they have a dedicated school health nurses. The ONMSD [12] acknowledge the potential importance of schools nurses in influencing the current and future health of the school going population. However, their findings indicate “an imbalance in the activities undertaken by schools nurses, in that immunisations tend to dominate possibly at the expense of health promotion activities” [12]. While large clinical caseloads are adversely affecting delivery of valuable population health initiatives, Irish PHNs are open to redressing “the balance of their roles in this regard” [12, page  27].

In contrast, the school health service in Norway can be seen as a continuation of the clinics' services and has a focused remit in health promotive and preventative work. Unlike Ireland the Norwegian PHN has office hours at the school and is available for pupils, (primary and postprimary) school administration, and collaborators at certain times of the week. Borup and Holstein's [77] Danish study concludes that school nurses play an important role for pupils in susceptible situations. However, Clausson's [78] doctoral thesis showed that PHNs have a deep knowledge of schoolchildren's health that is not used to its full potential. This finding indicates the difficulties in getting the model right in health systems that seem to have everything in terms of funding and policy commitment. This point is just as relevant to Ireland in the Celtic tiger era where there was money and a commitment to PHC [1] but reform was not delivered. 

It is much easier to support PHC reforms when growth in health expenditure is through prepaid systems than out of pocket expenditures [2]. Even though the Norwegian population enjoys good health, inequalities continue to exist in certain social groups [35, 37]. Norway's strategy to tackle social inequalities in health is to address the root causes of these inequalities. The current policy is geared specifically towards parts of the population where both the challenges and potential for improvement are greatest [79]. Equity is a specific goal that is top-down and government owned. The underpinning concept is a move from a health-specific to a coordinated strategy [17]. The strategy is to combine universal measures and general welfare with strategies that target the most vulnerable [80]. Coordination of services can, however, be time consuming and provide new challenges for PHNs regarding professional boundaries and co-location of services. Outcomes of collaboration can also be difficult to measure. 

## 6. Conclusion

Ireland and Norway have many similarities from a geographic and demographic perspective. Both countries have similar sized populations, but economically there are vast differences in relation to poverty, life expectancy that is lower, and inequalities that are higher in Ireland. A fundamental feature of primary care relates to equity of access to health services at the point of contact for all. However, health services are more accessible to high income earners in Ireland but universal health care is proposed. Differences identified relate to policy, economics, and public health achievements. A commitment to primary care in the view of the authors requires that health services be available free at the point of access. In the case of Ireland this will require a fundamental societal shift demanding a reexamination of the concept of equality and openness to higher taxation to fund health services. Nevertheless, both countries have a strong commitment to WHO reforms towards primary care, and PHNs have been identified as key players in the delivery of PC services, particularly primary prevention. The Norwegian PHN service model is specialist and aligned with a public health agenda. Ireland has been generalist to date but there is evidence of some movement in a specialist direction. On a very basic level, Norway has far more PHNs devoted specifically to public health issues, with one client group, compared with PHNs in Ireland providing services to all client groups with a preventative and curative remit. While Norway is a wealthy country and has realised an enviable PHN model, Ireland failed to achieve that and deliver on primary care reform, when money was available. Strategy embedded in public health policy similar to Norway is necessary to ensure that Public Health Nursing in Ireland is aligned with a public health agenda. It is, however, important to remember that despite Norway's wealth and specialist PHN model, everything is not perfect and current reforms may not provide the answer to complex problems. To quote the WHO (2008) [2, page  viii] “in moving forward, it is important to learn from the past and, in looking back, it is clear that we can do better in the future.”

## Figures and Tables

**Table 1:** Demography and vital statistics for Ireland and Norway.

	Ireland	Norway
Population	4,588,252 million	4.8 million
Over 65 years	535,393	742,000
Life expectancy	Female: 81.6 years Male: 76.8 years	Females: 83.4 years Males: 79 years
Birth rates	15.81 births/1,000 population	12.1 births/1,000 population
Infant mortality	3.2/1000 live births	3.5/1000 live births
Under 20 years	27.5%	26%
Density of population	64.95 people per square km	15 inhabitants per square km
Health spending	8.7% of GDP (under OECD average 8.9)	10% of GDP (over OECD average)

**Table 2:** Public health nursing: key similarities and differences between Ireland and Norway.

	Education	Organisational model	Remit	Focus of care	Challenges
Norway	1-year level 9 university postgraduate programme or 2 year Master programme	Decentralised to municipal level	Children, young people, and families	Prevention and promotion Egalitarian provision of and access to services Geographically based	Geographical conditions Issues of invisibility due to professional neutrality Funding Organisational model Implementation of the new coordination reform
Ireland	1-year level 9 university postgraduate programme	Employed by the health services executive and geographically based	All age groups (cradle to grave) regulated by the department of health policy	Preventative and curative Generalist and geographically based home visiting	Historical influences economic Organisational model Reform is overdue
